# Robust Learning-Based Detection with Cost Control and Byzantine Mitigation

**DOI:** 10.3390/s26010005

**Published:** 2025-12-19

**Authors:** Chen Zhong, M. Cenk Gursoy, Senem Velipasalar

**Affiliations:** Department of Electrical Engineering and Computer Science, Syracuse University, Syracuse, NY 13244, USA; czhong.ee@gmail.com (C.Z.); svelipas@syr.edu (S.V.)

**Keywords:** detection, controlled sensing, reinforcement learning, soft actor–critic algorithm, GAN, Byzantine attack

## Abstract

To address the state estimation and detection problem in the presence of noisy sensor observations, probing costs, and communication noise, we in this paper propose a soft actor-critic (SAC) deep reinforcement learning (DRL) framework for dynamically scheduling sensors and sequentially probing the state of a stochastic system. Moreover, considering Byzantine attacks, we design a generative adversarial network (GAN)-based framework to identify the Byzantine sensors. The GAN-based Byzantine detector and SAC-DRL-based agent are developed to operate in coordination to detect the state of the system reliably and fast while incurring small sensing cost. To evaluate the proposed framework, we measure the performance in terms of detection accuracy, stopping time, and the total probing cost needed for detection. Via simulation results, we analyze the performances and demonstrate that soft actor–critic algorithms are flexible and effective in action selection in imperfectly known environments due to the maximum entropy strategy and they can achieve stable performance levels in challenging test cases (e.g., involving jamming attacks, imperfectly known noise power levels, and high sensing cost scenarios). We also provide comparisons between the performances of the proposed soft actor–critic and conventional actor–critic algorithms as well as fixed scheduling strategies. Finally, we analyze the impact of Byzantine attacks and identify the reliability and accuracy improvements achieved by the GAN-based approach when combined with the SAC-DRL-based decision-making agent.

## 1. Introduction

State estimation/detection is critical in different applications, involving, for instance, remote health monitoring [1], smart grid [2], assembly lines, structural health monitoring, autonomous systems [3], adaptive radar [4], cognitive radio networks [5], and the Internet of Things (IoT). In such applications, it is important to monitor systems via sensors, and make reliable and time-sensitive decisions and detect anomalies (e.g., in order to maintain safe operation, identify faulty or compromised components, detect targets or obstacles, avoid collisions, and protect incumbent users).

Typically, detection is performed with noisy observations from the sensors. In this work, we consider two types of noise: the noise introduced by the sensors during sensing, and the noise in the communication links. The sensing noise affects the quality of sensing, and lower sensing noise generally indicates a more expensive sensor with a higher sensing cost. Noise in communication can be due to distortion in reception, interference, and/or adversarial jamming attacks. In this work, we seek a framework to learn the states and detect anomalies fast and accurately with potential cost control in the presence of adversarial attacks.

Specifically, we consider *N* sensors with different noise levels and different probing costs. The decision-maker dynamically selects sensors to collect the observations. Such a problem can be formulated within the framework of active hypothesis testing with cost control. The active hypothesis testing problem was first studied in [6]. Recently, more complicated and practical detection problems have been addressed. For instance, it is assumed that the prior information on the hypotheses is not perfectly known to the decision maker in [7], and the distribution of the observations is not distinguishable under some of the experiments in [8,9]. Extensions have also been explored to seek for a stopping rule that can hold in general cases [10]. Furthermore, similar to our purpose, the authors in [11] jointly considered the detection errors and the switching costs. Focusing on the constraints in the control problems, there are several algorithms developed based on the actor–critic algorithm [12] and soft actor–critic algorithm [13] for constrained Markov decision processes in recent works.

In the literature, various machine learning-based methods have been applied to address detection and hypothesis testing problems. For instance, learning approaches include the deep Q-network (DQN) [14,15], adversarial statistical learning [16], and deep actor–critic reinforcement learning [17]. In particular, the study in [18] addressed anomaly detection and developed sequential sensor selection policies (for monitoring multiple stochastic processes) using DQN, actor–critic, and active inference algorithms. In [19], scalable and decentralized algorithms were studied for sequentially selecting and observing stochastic processes, and deep actor–critic reinforcement learning framework was utilized. The authors in [20] considered a decentralized formulation of active hypothesis testing with multiple agents collecting noisy observations, and developed a multi-agent framework leveraging the actor–critic deep reinforcement learning approach. In [21], active sequential hypothesis testing in completely unknown environments was studied, and a combination of deep reinforcement learning and recurrent neural networks was proposed to address the problem. In [22], the authors analyzed target tracking with controlled sensing and derived theoretical properties of the optimal reinforcement learning policy. Compared with these methods, the recently proposed soft actor–critic reinforcement learning algorithm [23] exhibits advantages in exploring the unknown/uncertain environments due to the fact that the soft actor–critic (SAC) reinforcement learning algorithm is based on the maximum entropy reinforcement learning framework which encourages more evenly distributed probabilities for all actions and attempts to find a balance between exploration and exploitation. Motivated by this, we in this work propose a SAC-based decision-making agent for detection [24].

Moreover, we also consider the Byzantine attacks on the sensors. If the sensors are compromised, they become Byzantines which always quantize the signals to wrong states and send the distorted samples to the decision-maker. Conventionally, the Byzantine can be identified using Monte-Carlo methods [25]. However, in our setting, both the state of the target process and the state of the sensors may change before sufficient samples can be collected. Inspired by the application of generative adversarial networks (GANs) in detecting the changes in the statistics of time series data [26,27], we propose a GAN-based detector to identify the Byzantine sensors [24].

As noted above, there has been growing interest in leveraging machine learning methods for sequential decision-making in controlled sensing and active hypothesis testing problems. However, prior studies considered primarily DQN and actor–critic reinforcement algorithms and have not addressed the variations in the environment (e.g., in the form of increased communication noise due to, for instance, jamming attacks) and how the maximum entropy reinforcement learning framework can be utilized to adapt to uncertainty more effectively. Moreover, the presence of Byzantine sensors and the detection of adversarial attacks have not been considered in the aforementioned studies on controlled sensing. Motivated by these gaps, we provide the following contributions in this paper:

- We design a soft actor–critic (SAC) deep reinforcement learning (DRL)-based algorithm for sequential sensor scheduling for detecting the state of a stochastic process. We consider a general setting with *M* states, *N* sensors with different costs, and sensing and communication noise.
- In the design of the SAC-DRL algorithm, we consider both log-likelihood and entropy-based rewards and take into account the cost of sensing. The SAC-DRL agent is developed with the goal to detect the state of the process reliably (with confidence exceeding a given threshold) and quickly at small sensing cost.
- We address the presence of compromised Byzantine sensors that feed wrong results to the decision-making agent. We develop a GAN-based approach to detect these Byzantine sensors and eliminate their observations in order to improve the accuracy of the detection results. GAN-based detector and SAC-DRL-based agent operate in coordination to provide reliable decision-making.
- We conduct extensive simulations and evaluate the performance of baseline strategies (including fixed sensor scheduling, conventional actor–critic algorithm) and the proposed SAC-DRL algorithm in terms of detection accuracy, stopping time, and sensing cost. We analyze the impact of jamming attacks and increased (unknown) communication noise power as well as Byzantine attacks on sensors. We demonstrate the effectiveness of the proposed framework of a combined GAN-based Byzantine detector and SAC-DRL-based decision-maker in challenging practical environments.

The remainder of the paper is organized as follows. The system model is described in Section 2 and the problem formulation is provided in Section 3. In Section 4, we design the learning-based decision-maker and the Byzantine detector. In Section 5, experimental results are presented and analyzed. Finally, the conclusions are drawn in Section 6.

## 2. System Model

We consider a scenario in which there are *N* remote sensors monitoring a target process. The state of the process can switch between *M* possible states. Here, we assume that the state of the process can be denoted as a signal $S\in \{s_{1},\ldots ,s_{m},\ldots ,s_{M}\}$ and each element $s_{m}$ (m=1,2,…,M) stands for a possible state. We consider that a decision-making agent dynamically selects the sensors to probe the process state and makes decisions on the process state based on the samples collected by all the selected sensors. When the selected sensors probe the process state, the corresponding state signal $S=s_{m}$ will be observed by every probing sensor albeit with noise. The sensors estimate the process state and report to the decision-maker individually.

**Figure 1:**
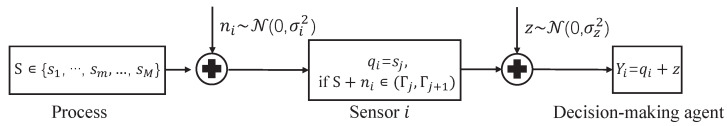
A diagram that depicts the noisy observations of a single sensor and the noisy observations of the decision-maker.

Ideally, the process state *S* can be detected using only one sample. In practice, however, the sensor observations and/or the communication links are noisy. Hence, the decision-maker needs to observe multiple samples to ensure detection accuracy. A diagram of the sensing and transmission by a single sensor in the presence of noise is depicted in Figure 1. Due to their types and differences, sensors experience different levels of noise in their observations. The noise introduced in the observation of sensor *i* is modeled as Gaussian with zero mean and variance $\sigma_{i}^{2}$, i.e.,

(1) $$\begin{array}{c} n_{i}∼N\left(0,\sigma_{i}^{2}\right). \end{array}$$

Therefore, the received sensing/observation signal at sensor *i* can be expressed as $s+n_{i}$. We also assume that, at each time when sensor *i* requests a state signal, there is a probing cost $c_{i}$. Furthermore, for the sensor whose noise power $\sigma_{i}^{2}$ is lower, the corresponding cost $c_{i}$ is typically higher. After receiving the observation, the sensors quantize the signal according to a set of predefined thresholds $\left\{\Gamma_{1},\Gamma_{2},\ldots ,\Gamma_{M+1}\right\}$. We assume

(2) $$\begin{array}{c} -\infty =\Gamma_{1}<s_{1}<\Gamma_{2}<\cdots <s_{m}<\Gamma_{m+1}<\cdots <s_{M}<\Gamma_{M+1}=\infty , \end{array}$$

and the sensor *i* quantizes the signal as

(3) $$\begin{array}{c} q_{i}=s_{j},\mathrm{if}\;S+n_{i}\in \left(\Gamma_{j},\Gamma_{j+1}\right),\exists j\in \left\{1,2,\ldots ,M+1\right\}. \end{array}$$

Then the sensor *i* transmits the quantized signal $q_{i}$ to the decision-maker over a communication channel. Reception over the channel is distorted by another additive Gaussian noise *z* with mean zero and variance $\sigma_{z}^{2}$, i.e.,

(4) $$\begin{array}{c} z∼N(0,\sigma_{z}^{2}). \end{array}$$

Therefore, the signal received at the decision-maker from sensor *i* is denoted as

(5) $$\begin{array}{c} Y=q_{i}+z. \end{array}$$

In the considered setting with noisy observations, we propose a soft actor–critic based decision-making agent that aims at dynamically selecting sensors in order to make a decision quickly with a certain confidence level at a small sensing cost. Here, while we have assumed without loss of generality that sensed signals directly match the values of the process states, the analysis is general and applicable to any other fixed signaling values that represent different states. Furthermore, the system model can also be extended to cases with multiple processes and multiple states for each process.

## 3. Problem Formulation

### 3.1. Stopping Rule

Since the process has *M* possible states, we have *M* hypotheses. We denote the prior probabilities of each hypothesis being true by the probability vector $\pi =[\pi_{1},\pi_{2},\ldots ,\pi_{M}]$. With this, we further denote by $\pi_{m}^{t}$ the posterior belief of the hypothesis $H_{m}$ being true at time *t*, and express the posterior belief as

(6) $$\pi_{m}^{t}=\mathrm{Pr}\left\{S=s_{m}|Y_{1},\ldots ,Y_{t}\right\}=\frac{\pi_{m}\prod_{\tau =1}^{t}p\left(Y_{\tau }|S=s_{m}\right)}{\sum_{l=1}^{M}\pi_{l}\prod_{\tau =1}^{t}p\left(Y_{\tau }|S=s_{l}\right)}$$

where $p(Y_{t}|S=s_{m})$ is the distribution of $Y_{t}$ observed by the decision-maker at time *t* given that the process state is $S=s_{m}$, and this distribution is derived below. We note that when the agent selects to observe *n* processes, n=1,…,N, in a time slot, the posterior probabilities will be updated *n* times.

The conditional distribution $p(Y|S=s_{m})$ can be expressed as

(7)p(Y|S=sm)=∑l=1Mp(Y,q=sl|S=sm)(8)            =∑l=1MP(q=sl|S=sm)p(Y|q=sl,S=sm).

Since the variables *Y* and *S* are conditionally independent, (8) can be rewritten as

(9) $$\begin{array}{c} p\left(Y|S=s_{m}\right)=\sum_{l=1}^{M}P\left(q=s_{l}|S=s_{m}\right)p\left(Y|q=s_{l}\right) \end{array}$$

The sensors apply the detection rule in (3), and therefore the conditional probabilities P(q|S) can be expressed in terms of the Gaussian *Q* function. For instance,

(10) $$\begin{array}{c} p\left(q=s_{j}|S=s_{m}\right)=p\left(\Gamma_{j}-s_{m}<n_{i}<\Gamma_{j+1}-s_{m}\right)=Q\left(\frac{\Gamma_{j}-s_{m}}{\sigma_{i}}\right)-Q\left(\frac{\Gamma_{j+1}-s_{m}}{\sigma_{i}}\right). \end{array}$$

The conditional distribution $p(Y|q=s_{j})$ is Gaussian with mean $s_{j}$ and variance $\sigma_{z}^{2}$, i.e., we have

(11) $$\begin{array}{c} p\left(Y|q=s_{j}\right)=N\left(s_{j},\sigma_{z}^{2}\right). \end{array}$$

Then, substituting (10) and (11) into (8), we can obtain the conditional probability density function of *Y* given the source signal *S*, and utilize it to update the posterior probability in (6). We note that the above formulations on the conditional distributions can be adapted to noise or distortion models with non-Gaussian distributions, and once the posterior probabilities are computed, the remainder of the analysis and algorithm development would proceed in essentially the same manner.

**Figure 2:**
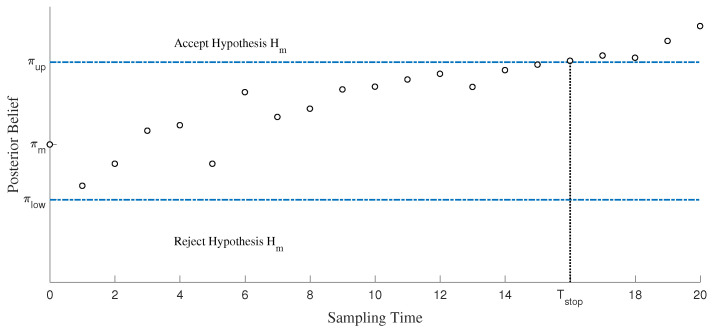
An example of stopping time.

As shown in Figure 2, the hypothesis $H_{m}$ is claimed to be accepted when the posterior belief $\pi_{m}$ is greater than the upper bound $\pi_{\mathrm{upper}}$, or to be rejected when the posterior belief is less than the lower bound $\pi_{\mathrm{lower}}$. Furthermore, once any of the *M* hypotheses is accepted, the observer stops receiving samples immediately.

### 3.2. Confidence Measures and Rewards

In this work, we consider two different confidence measures, and we derive two rewards based on them to be used in the learning algorithms.

#### 3.2.1. Log-Likelihood Ratio-Based Reward

Similarly as in [14,28], we consider the confidence level as the maximization objective. One confidence measure in terms of the posterior probability at time *t* is given by the average Bayesian log-likelihood ratio (LLR):

(12) $$C\left(\pi \left(t\right)\right)=\sum_{m=1}^{M}\pi_{m}^{t}\log\frac{\pi_{m}^{t}}{1-\pi_{m}^{t}}.$$

Correspondingly, the LLR-based reward, which measures the improvement in the confidence level from time t−1 to time *t*, is defined as

(13) $$r_{C}\left(t\right)=C\left(\pi \left(t\right)\right)-C\left(\pi \left(t-1\right)\right).$$

#### 3.2.2. Entropy-Based Reward

Confidence can also be measured via the entropy. Since the entropy of the posterior probability distribution is minimized by having one of the posterior probabilities be 1 and all the other probabilities be 0, we can also consider an entropy-based reward given as

(14) $$r_{H}\left(t\right)=H\left(\pi \left(t-1\right)\right)-H\left(\pi \left(t\right)\right)$$

where entropy is formulated as

(15) $$H\left(\pi \left(t\right)\right)=-\sum_{m=1}^{M}\pi_{m}^{t}\log\pi_{m}^{t}.$$

### 3.3. Cost

As mentioned in the previous section, we consider a sensing cost and incorporate this cost into the reward function (as described in (18) below). This instantaneous cost c(t) depends on the cost of the sensors that are selected in time slot *t*. More specifically, we define the cost at time *t* as follows:

(16) $$c\left(t\right)=\left\{\begin{array}{c} \lambda \sum_{i}\left(1\left(i\right)\cdot c_{i}\right)\;\;\;\;\;\;\mathrm{without}\;a\;\mathrm{designed}\;\mathrm{target}\;\mathrm{average}\;\mathrm{cost},\\ \lambda |\bar{c}-\sum_{i}\left(1\left(i\right)\cdot c_{i}\right)|\;\;\;\;\;\mathrm{with}\;a\;\mathrm{designed}\;\mathrm{target}\;\mathrm{average}\;\mathrm{cost}\;\bar{c} \end{array}\right.$$

where 1(i)∈{0,1} indicates whether sensor *i* is selected at time *t*, $c_{i}$ is the cost of using sensor *i*, λ is a predefined weight factor to control the influence of the cost on the reward function, and $\bar{c}$ ($0<\bar{c}<\sum_{i}c_{i}$) denotes the predefined average cost that the agent targets. That is to say, the predefined average cost is considered as a soft constraint, and the agent aims at fully utilizing the budget but not exceeding it.

## 4. Learning-Based Solutions

### 4.1. Workflow

**Figure 3:**
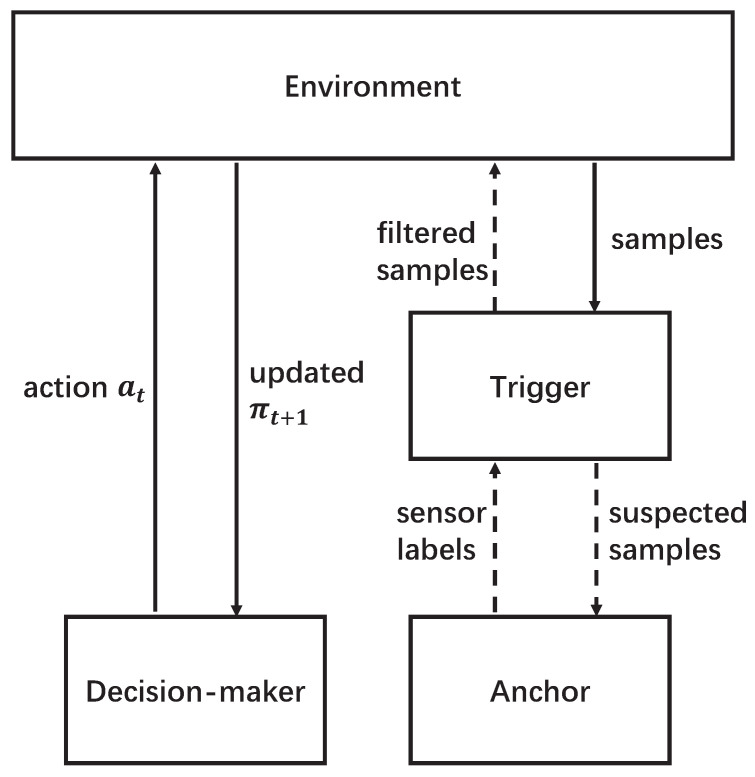
Workflow of the learning-based detection scheme.

To handle the hypothesis testing problem, and jointly control the potential risks during the detection, we in this work propose the detection scheme shown in Figure 3. The detection scheme consists of three parts: the environment, a decision-maker, and a Byzantine detector that includes a trigger and an anchor node.

*Environment:* The environment consists of the process and all the sensors. In the environment, the samples and the feedback after executing the actions selected by the decision-maker can be dynamically generated, and the state of the process updates at the beginning of each episode.

*Decision-maker:* Based on the observations, the decision-maker is responsible for dynamically selecting sensors to sequentially probe the process and for terminating the probing when the confidence level exceeds a predefined threshold and for detecting the state of the process. The decision-maker is based on the soft actor–critic reinforcement learning algorithm, and it aims to detect the process state as accurately as possible while controlling the probing cost. Furthermore, the algorithm is supposed to be able to work robustly in the presence of additional uncertainty that can be caused by increased noise/interference (e.g., due to jamming attacks).

*Trigger and Anchor Nodes:* The trigger and anchor nodes are employed as the Byzantine detector when there are potential Byzantine sensors in the system. The two parts are designed to identify the Byzantines and eliminate the samples collected by those sensors. Specifically, the trigger will first inspect every newly collected sample and report the suspected samples to the anchor node. Then, the anchor node will compare the suspected samples with the samples that are collected by the anchor node. Here, we assume that the anchor node is reliable and, due to its high reliability, the probing cost of the anchor node is very high. Therefore, the anchor node is not used for probing the process, and there is a trigger employed to reduce the usage of the anchor node in identifying the Byzantines. In this work, since the distribution of the process states and the sensors’ information are unknown to the agents, and the number of samples is limited, we apply the GAN algorithm to reconstruct the distribution of samples in each state and take advantage of the reconstructed features to identify the Byzantines.

### 4.2. Decision-Maker: SAC-Based Agent

In this section, we describe the proposed soft actor–critic learning framework for the considered detection problem.

#### 4.2.1. Preliminaries

We first introduce the relevant definitions within the framework.

*Agent’s Observation and State:* Since the agent can only have observations from the selected sensors/processes, the problem can be modeled as a partially observable Markov decision process (POMDP). With the given observations, the agent can update the posterior belief $\pi^{t}$ according to (6). Furthermore, we take the reward $r_{t}$ obtained by the selected action $a_{i}$ as the state (or input) of the agent, and we denote the state at time *t* as $O_{t}$. The state $O_{t}$ is a 1×M vector, and each element $O_{t,i}$ denotes the observation obtained by taking the action $a_{i}$ at time *t*, which is defined as follows:

(17) $$O_{t,i}=\left\{\begin{array}{c} r_{t}\;\mathrm{if}\;\mathrm{action}\;a_{i}\;\mathrm{is}\;\mathrm{selected}\;\mathrm{at}\;\mathrm{time}\;t\\ 0\;\mathrm{otherwise} \end{array}\right..$$

The definitions of action $a_{i}$ and reward $r_{t}$ are introduced below. Furthermore, we assume that the agent can keep at most M latest observations.

*Action:* We denote the action space as A, in which all valid actions are included. Since the agent can select any combination of *k* sensors at a time (k∈{1,2,…,N}), the size of the action space is ∑k=1NNk, and a valid action *a* stands for selecting the corresponding sensors and receiving the samples to update the posterior belief. In each iteration, the agent will estimate the probability distribution of selecting each valid action and choose one action to execute according to this estimated distribution .

*Reward:* Since the decision-making agent aims to reach the required confidence level as soon as possible while incurring small sensing cost, it should maximize the accumulated reward from the first time slot to the stopping time $T_{\mathrm{stop}}$ in an episode. So we define the immediate reward $r_{t}$ as

(18) $$r_{t}=\left\{\begin{array}{c} r_{C}\left(t\right)-c\left(t\right)\;\mathrm{if}\;\mathrm{LLR}\text{-}\mathrm{based}\;\mathrm{reward}\;\mathrm{is}\;\mathrm{employed}\\ r_{H}\left(t\right)-c\left(t\right)\;\mathrm{if}\;\mathrm{entropy}\text{-}\mathrm{based}\;\mathrm{reward}\;\mathrm{is}\;\mathrm{employed} \end{array}\right.$$

and the accumulated reward is expressed as

(19) $$r_{1:T}=\left\{\begin{array}{c} C\left(\pi \left(T\right)\right)-C\left(\pi \left(1\right)\right)-\sum_{t=1}^{T}c\left(t\right)\;\mathrm{LLR}\text{-}\mathrm{based}\;\mathrm{reward}\\ H\left(\pi \left(1\right)\right)-H\left(\pi \left(T\right)\right)-\sum_{t=1}^{T}c\left(t\right)\;\mathrm{entropy}\text{-}\mathrm{based}\;\mathrm{reward} \end{array}\right.$$

Here, we define the state $O_{T}$ as the terminal state if any of the *M* hypotheses is claimed to be accepted, i.e., $\max\left(\pi^{T-1}\right)\geq \pi_{\mathrm{upper}}$, where $\pi_{\mathrm{upper}}$ is the predefined upper threshold used to declare acceptance of a hypothesis. When we update the agent, we consider a weighted reward $R_{t}$ at time t≤T, as a discounted sum of the rewards:

(20) $$R_{t}=\sum_{\tau =t}^{T}\eta^{\tau -t}\;r_{\tau },$$

so that actions leading to better future outcomes receive higher returns. In the implementation, the agent will be updated *T* times after the terminal state has been reached, using the weighted reward achieved at the terminal time *T*, and all the way back to the initial time t=0.

#### 4.2.2. Soft Actor–Critic Algorithm

In this subsection, we describe the architecture of the soft actor–critic algorithm. The soft actor–critic architecture consists of three neural networks: policy network, Q network, and value network. These three networks will not share any neurons but exchange information to update each other.

*Policy network:* The policy network is employed to explore a policy μ that maps the agent’s observation O to the action space A:

(21) $$\mu_{\phi }\left(O\right):O\to A.$$

So the mapping policy $\mu_{\phi }\left(O\right)$ is a function of the observation O and is parameterized by ϕ. The chosen action can be denoted as

(22) $$a=\mu_{\phi }\left(O\right)$$

where we have a∈A. Since the action space is discrete, we use the softmax function at the output layer of the policy network so that we can obtain the scores of each action. The scores sum up to 1 and can be regarded as the probabilities of obtaining a good reward when the corresponding actions are chosen.

*Q network:* The Q network $Q_{\theta }$, parameterized by θ, is an approximator to the soft Q function. It is fed the (O,a) pairs, and it estimates the corresponding Q value. The Q network encourages the policy to converge to the real Q value distribution instead of converging to a promising action. In this way, the agent tends to explore the environment more and engage in effective exploration strategies.

*Value network:* The value network $V_{\psi }\left(O\right)$ is parameterized by ψ, and it estimates the soft values of the given states. Since the estimated state value indicates the potential future reward, the value network encourages the policy to exploit the promising actions that are learned from the experience.

*Update:* To update the neural networks, we adopt a memory D to store the historical transitions and sample a minibatch at every iteration. All three neural networks are updated using stochastic gradient descent.

The value network is updated by minimizing the squared residual error as follows:

(23) $$J_{V_{\psi }}=E_{O_{t}∼D}\left[\frac{1}{2}{\left(V_{\psi }\left(O_{t}\right)-E_{a_{t}∼\mu_{\phi }}\left[Q_{\theta }\left(O_{t},a_{t}\right)-\log\mu_{\phi }\left(a_{t}|O_{t}\right)\right]\right)}^{2}\right].$$

The Q network is updated by minimizing the soft Bellman residual as follows:

(24) $$J_{Q_{\theta }}=E_{(O_{t},a_{t})∼D}\left[\frac{1}{2}{\left(Q_{\theta }\left(O_{t},a_{t}\right)-{\hat{Q}}_{\theta }\left(O_{t},a_{t}\right)\right)}^{2}\right]$$

where ${\hat{Q}}_{\theta }\left(O_{t},a_{t}\right)=r\left(O_{t},a_{t}\right)-c_{t}+\gamma E\left[V_{\psi }\left(O_{t+1}\right)\right]$.

The policy network is trained by minimizing the expected KL divergence

(25) $$J_{\mu_{\phi }}=E_{O_{t}∼D}[D_{KL}(\mu_{\phi }\left(\cdot |O_{t}\right)||\mathrm{softmax}\left(Q_{\theta }\left(O_{t},\cdot \right)\right))].$$

The full framework is described in Algorithm 1 below.
**Algorithm 1** Soft Actor–Critic Algorithm for Dynamic Sensor Selection  1:t=0  2:Initialize the value network $V_{\psi }\left(O\right)$, Q network $Q_{\theta }\left(O,a\right)$ and the policy network $\mu_{\psi }\left(O\right)$, parameterized by ψ, θ and ϕ, respectively.  3:The agent initializes the memory D.  4:**for**doT=1:Maximumepisode  5:    $t_{\mathrm{start}}=t$  6:    Generate a new hypothesis $H_{j}$ to be true according to the prior belief π, and j∈{1,…,M}.  7:    The agent fetches the prior belief vector π as the initial state.  8:    **while** $\;\mathrm{do}O_{T}$ is not a terminal state  9:        t←t+110:        With the state $O_{t}$, the agent selects the sensors (out of the *N* sensors) according to the decision policy $a_{t}=\mu_{\phi }\left(O_{t}\right)$ with respect to the current policy.11:        Agent receives the samples $Y_{t}$ from each chosen sensor and updates the posterior belief vector $\pi^{t}$.12:        With the new state $O_{t+1}$, the agent obtains a reward $r_{t}$ and cost $c_{t}$.13:        Update the state $O_{t}=O_{t+1}$.14:    **end while**15:    R=016:    **for** $\;\mathrm{do}\tau =t-1:t_{\mathrm{start}}$17:        $R\leftarrow r_{\tau }+\eta *R$18:        Update the neural networks according to Equations (23)–(25).19:    **end for**20:    Reveal the true hypothesis, and check the accuracy of detection.21:**end for**22:Save the trained neural networks.

### 4.3. Trigger and Anchor: GAN-Based Byzantine Detector

In this section, we consider the scenario that the decision-maker is operating in the presence of Byzantine attacks. It is assumed that when a sensor is under attack, this sensor becomes a Byzantine. Furthermore, it is also assumed that the number of Byzantines, k∈{0,1,…,N} is unknown to the decision-maker. Same as the honest sensors, the Byzantine sensors quantize the samples using the thresholds in (2). However, the Byzantine sensors always flip the samples after quantization according to a pattern. For instance, when the quantized sample is $s_{i}$, the Byzantine sensor will send $s_{j}$ to the decision-maker, and if the quantized sample is $s_{j}$, the Byzantine sensor will send $s_{i}$ to the decision-maker. Here, we have i,j∈{1,2,…,M} and i≠j.

Ideally, since all the sensors are probing the same process, the samples collected by the sensors should be the same in the absence of noise. Therefore, compared to the sample sent by the anchor node, the sensors that send different signals are the Byzantines. However, we consider both the sensor noise and the noise in transmission channels in this work. Such noise makes the samples collected by each sensor be distributed with different variances. The mean values will also be different when Byzantines send different signals. Hence, we need to estimate the mean value of the samples before comparing with samples from the anchor node. To obtain reliable mean values, a large number of samples may be needed. In practice, the posterior probability may reach the stopping criteria before the decision-maker collects sufficient samples. To solve this problem, we propose a GAN-based detector to estimate the mean values using a single sample.

#### 4.3.1. Trigger and Anchor Node

**Figure 4:**
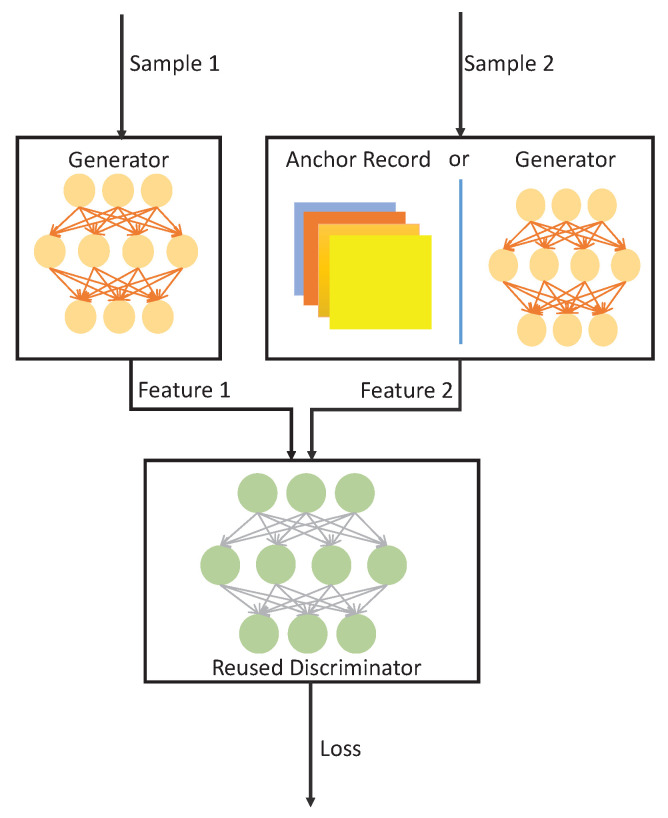
Structure of the GAN-based Byzantine detector.

The Byzantine detector consists of two parts: a trigger and an anchor node. The trigger is modeled as a GAN. The anchor node also operates with another GAN. The structure of the GAN-based models is depicted in Figure 4. There are two samples that are collected by different nodes to be compared using the GAN. Here, the input “sample 1” comes from the suspected sensor, and the input “sample 2” comes from the reference sensor or the anchor node as discussed below.

At each step, the trigger is employed to screen all the samples and report the suspected samples. Once a new sample is collected, the corresponding sensor will be considered as a potential suspect sensor and all the other sensors will be the reference sensors. The features of “sample 1” (which come from the potential suspect sensor) and “sample 2” (which come from a reference sensor) will be reconstructed by the generator. Then, the two features will be the inputs of the discriminator to calculate a loss between these two samples. We assume that in each episode, the GAN can keep a record of B latest samples from each sensor. Hence, the latest sample of each potential suspect sensor will be compared with at most B(N−1) samples from the reference sensors, and after each comparison, a corresponding loss will be obtained. Based on all these losses, the GAN will decide on whether “sample 1” is indeed a suspected sample.

As shown in Figure 3, only if a sample is confirmed and labeled as a suspected sample, will it be sent to the anchor node and its associated GAN to be the input “sample 1”. Furthermore, once the suspected sample is received, the anchor node will be triggered and take one sample on the process, and this sample will be the input “sample 2” in the GAN. It is assumed that the anchor node is reliable and the GAN has already collected a record of the sample features during the training process. Therefore, the features of “sample 1” are reconstructed using the generator, and the features of “sample 2” will be extracted from the anchor record. Similar to the trigger, a loss between the two samples will be calculated and the final decision on whether the corresponding sensor is a Byzantine will be made. Once a sensor is identified as a Byzantine, the samples collected by it will be eliminated.

#### 4.3.2. GAN

The general structure of a GAN is introduced in [29]. A GAN consists of two neural networks: a generator *G* that is used to capture the statistical features of the data and a discriminator *D* that is used to estimate the probability that a sample comes from the training data rather than the generative model *G* to evaluate the generative policy.

We first define a sample space S with a probability distribution p(s|x), where s is a set of samples corresponding to the real data x in the training data set. The generator *G* maps the sample into the real data space:

(26) $$G\left(s;G\right):s\to \hat{x},$$

where G denotes the parameters of the generator neural network, and the $\hat{x}$ is a projection (of real data x) generated by the generator *G*.

The discriminator $D(\tilde{x};\omega )$ estimates the probability of the input $\tilde{x}$ coming from the real data set, where ω denotes the parameters of the discriminator neural network, and the input $\tilde{x}$ can be either the real data x or the generated data $\hat{x}$.

In the training phase, the GAN is trained to take samples generated according to both safe sensor data distribution ($z∼N(q_{i},\sigma_{z}^{2})$) and the attacked sensor data distribution ($z∼N(\tilde{q_{i}},\sigma_{z}^{2})$), where $\tilde{q_{i}}$ is the flipped sample when the actual signal is $q_{i}$. In particular, the generator aims at reconstructing the samples’ statistics and the discriminator compares the reconstructed statistics with the designed one to guide the generator. Specifically, in the testing phase, the GAN compares the statistics reconstructed from the samples collected by the sensors and the statistics provided by the anchor node and decides whether the samples collected by the sensors have the same statistics as the anchor node data.

A good discriminator *D* is expected to be able to distinguish the generated data from the real data, i.e., the estimated probability should be very small if the input is the generated data and should be close to 1 if the input is from the real data. Therefore, the discriminator aims at minimizing the objective function given as

(27) $$L_{D}=-\left(\log\left(D\left(x\right)\right)+\log\left(1-D\left(\hat{x}\right)\right)\right).$$

On the other hand, for a generator *G* that has the goal to learn the real data distribution, the evaluation $D(\hat{x})$ acts as guidance for the updates of the generative model. Thus, a good generator *G* should be able to make the generated data indistinguishable from the real data to the discriminator *D*. In addition, since the generator aims at reconstructing the mean of the samples, the difference in the mean of generated data and anchor data should be considered in the loss function. For this purpose, the generator *G* seeks to minimize the following objective function:

(28) $$L_{G}=\log\left(1-D\left(\hat{x}\right)\right)+w\cdot {\left(\mathrm{mean}\left(\hat{x}\right)-\mathrm{mean}\left(x\right)\right)}^{2}.$$

where *w* is the weight to rescale the difference in the mean of the corresponding data. The workflow of the GAN is presented in Algorithm 2 below.
**Algorithm 2** Workflow of GAN  1:Initialize the generator G(s;G) with the parameters G, and the the discriminator D(x;ω), parameterized by ω.  2:**for**doT=1:Maximumepisode  3:    Fetch the sample set s(t) and the corresponding real data set x(t).  4:    Use the generator *G* to generate the projection of the real data: $\hat{x}\left(t\right)=G\left(s\left(t\right);G\left(t\right)\right)$  5:    Feed the projection $\hat{x}\left(t\right)$ and the real data x(t) to the discriminator *D*, and obtain the estimated probabilities of both data being real data.  6:    Update the discriminator by descending the stochastic gradient:  7:    $-\nabla_{\omega }(\log(D\left(x\left(t\right);\omega \left(t\right)\right)+\log\left(1-D\left(\hat{x}\left(t\right);\omega \left(t\right)\right)\right)$  8:    Update the generator by descending the stochastic gradient:  9:    $\nabla_{G}\log(1-D\left(\hat{x}\left(t\right);\omega \left(t\right)\right)+w\cdot {\left(\mathrm{mean}\left(\hat{x}\right)-\mathrm{mean}\left(x\right)\right)}^{2}$10:**end for**

## 5. Simulation Results

### 5.1. Experimental Settings

#### 5.1.1. Environment

In the experiments, the target process has four possible states (i.e., M=4) and signal values for these states are denoted as 0, 0.25, 0.5, and 1. We set the number of sensors to be N=3, and the sensor noise power vector is [0.2,0.1,0.05] and the corresponding cost vector is [0.1,0.5,1]. Hence, sensors with smaller noise power have higher cost. The noise power in the communication links can be selected from the set {0.05,0.2,0.4,0.6}. Normally, the channel noise power is assumed to be known to the decision-maker in the absence of any interference sources. However, when the channel has interference from other transmitters or experiences jamming attacks, the actual channel noise power becomes unknown to the decision-maker. In this case, the decision-maker underestimates the noise power by assuming it to be equal to 0.05, and uses this incorrect noise power level to update the posterior probabilities $\pi^{t}$. For every episode, if a sensor becomes a Byzantine, that sensor will swap the signal among the two pairs: (0,0.5) and (0.25,1).

#### 5.1.2. Neural Networks

The configuration of the proposed soft actor–critic (SAC) framework is provided in Table 1. For comparison purposes, we also implement the conventional actor–critic (AC) algorithm. The configuration of the AC framework is provided in Table 2. The implementation of the AC framework is also explored in [30]. In the experiments, both the LLR-based reward and entropy-based reward will be considered for the actor–critic framework. The configuration of the GAN is provided in Table 3.

In general, the computational complexity of the SAC algorithm is higher than that of the conventional AC algorithm because the SAC framework involves more neural networks (e.g., policy, Q, and value networks) and has more computations per update (e.g., due to entropy regularization for which log probabilities are computed and sampling actions from stochastic policy are performed). On the other hand, the SAC algorithm has the benefits of improved sample efficiency and better exploration due to entropy maximization.

In the proposed approach, all possible combinations of the sensors are considered in the selection process. Correspondingly, the size of the action space is ∑k=1NNk=2N−1, which dictates the number of neurons in the output layers of the policy network in the SAC and the actor network in a conventional AC. Hence, as the number of sensors, *N*, increases, the complexity grows rapidly (requiring more computations and more training time). Consequently, for large *N*, limiting the number of sensors that can be selected at each time to a small value provides a useful reduction in complexity and improves scalability. For instance, as done in [19], if only one sensor is selected at each time, the action space has a size of *N*, resulting in only linear growth in the number of sensors. However, this generally incurs longer stopping times and increased detection delay.

### 5.2. Numerical Results

#### 5.2.1. Preliminary Results

**Figure 5:**
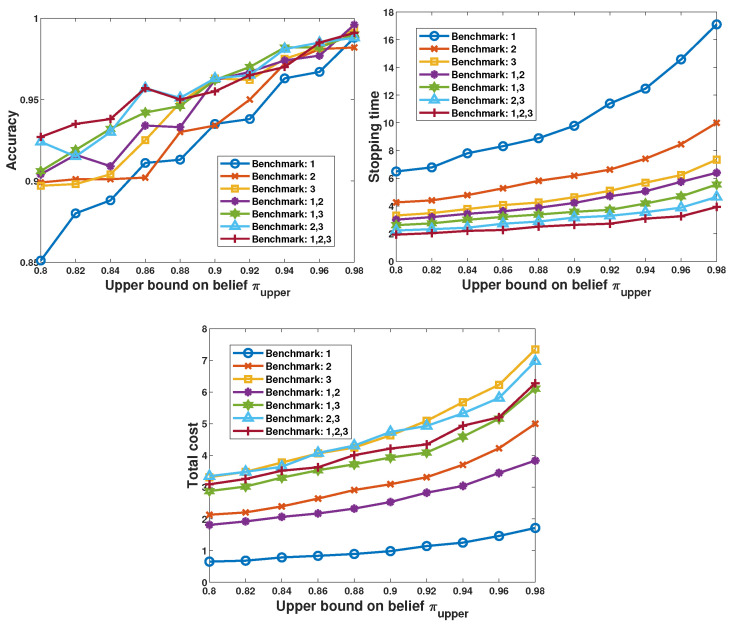
Performance of the benchmarks when $\pi_{\mathrm{upper}}$ is varied from 0.8 to 0.98, λ = 0, and $\sigma_{z}^{2}=0.05$.

To illustrate how the selection of sensors influences the performance of the decision-maker, we first investigate the naive fixed sensor-selection policies as a benchmark. The naive policies select their preferred sensors all the time. Since N=3, there are seven different naive policies, and each of them selects a different set of sensors: {{1}, {2}, {3}, {1,2}, {1,3}, {2,3}, {1,2,3}}. For instance, the policy with {1,2,3} selects all three sensors all the time. We consider the detection accuracy, average stopping time, and the average total cost over 10,000 episodes as the key performance metrics. In Figure 5, we set λ=0, and $\sigma_{z}^{2}=0.05$, and plot the performance metrics as a function of the confidence threshold $\pi_{\mathrm{upper}}$. As shown in the figure, all three performance metrics grow as the predefined confidence level increases. As noted before, sensor 1 has the highest noise power and the lowest cost. So, it can be observed that the policy “Benchmark:1” achieves the lowest accuracy and the lowest cost, but the highest stopping time. Furthermore, it can be observed that when the $\pi_{\mathrm{upper}}$ is low, the accuracy achieved by the policies whose selections include sensor 3 are relatively higher. Correspondingly, the stopping times are lower and the total costs are higher.

These observations indicate that to reach the stopping criteria, the decision-maker needs to obtain sufficient information on the sample distribution, and both the number of samples and the reliability of the selected sensors influence the performance. Therefore, the proposed learning-based decision-maker is expected to be able to distinguish the most reliable sensor from the feedback and control the probing cost.

**Figure 6:**
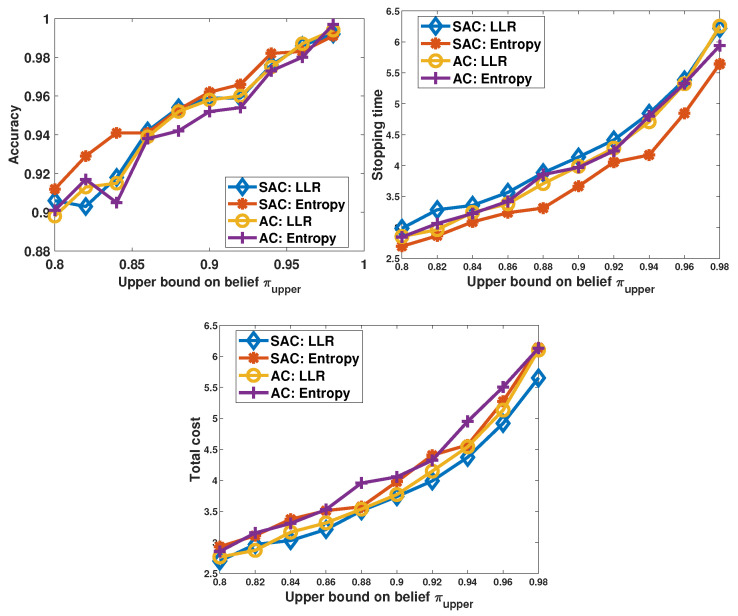
Performance of the learning-based decision-makers when $\pi_{\mathrm{upper}}$ is varied from 0.8 to 0.98, λ = 0, and $\sigma_{z}^{2}=0.05$.

In Figure 6, we illustrate the performance of the proposed SAC decision-maker, and we compare its performance with the AC-based decision-maker. Both DRL-based algorithms are tested with two types of reward functions: LLR and entropy. Generally, the performance of the DRL-based algorithms are competitive. When compared to the benchmarks, the DRL-based algorithms are similar to the “Benchmark:1,2” and “Benchmark:1,3”. For the SAC-based algorithms, the policy with the LLR reward performs better than the policy with the entropy reward in terms of the stopping time. For the AC algorithm, the policy with the LLR reward achieves a higher accuracy then the policy with the entropy reward. It can also be observed that for each type of reward, considering the three performance metrics, the SAC-based algorithms are slightly better than the AC-based algorithms.

#### 5.2.2. Jamming Attacks and Increased Noise Power

**Figure 7:**
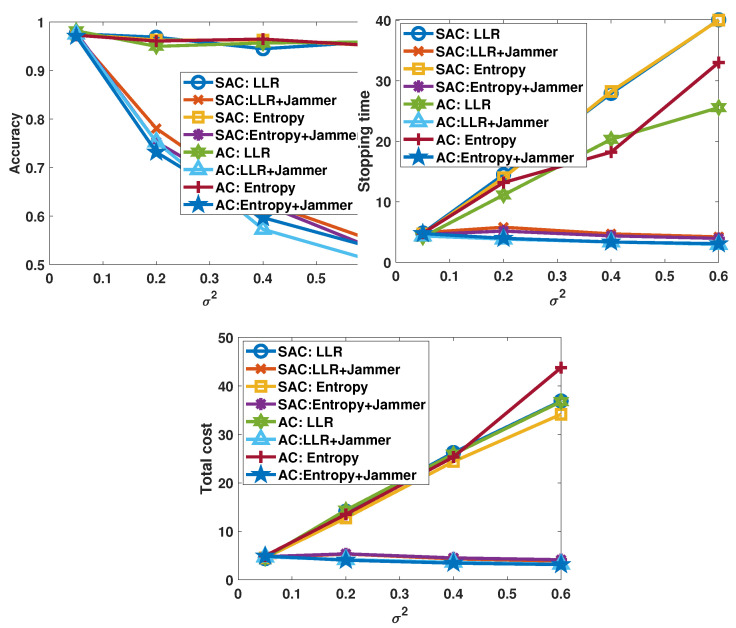
Performance of the learning-based decision-makers when the $\sigma^{2}$ varies from 0.05 to 0.6, λ = 0, and $\pi_{\mathrm{upper}}=0.94$.

We now consider the scenario in which the transmission channel is attacked by a jammer during the experiment and the actual channel noise $\sigma^{2}$ is unknown to the decision-maker. In this experiment, the actual channel noise power varies as $\sigma^{2}\in \left\{0.05,0.2,0.4,0.6\right\}$, but the decision-maker always uses $\sigma_{z}^{2}=0.05$ to update the posterior probabilities. In Figure 7, we plot the performance metrics as functions of the actual channel noise power $\sigma^{2}$. For all the decision-makers that we have tested, as $\sigma^{2}$ increases, the performance becomes worse: the accuracy decreases rapidly and both the stopping time and total cost decrease slightly (as seen by the curves labeled with “+Jammer”). The degraded performance can be attributed to the uncertainty introduced by the channel noise. Specifically, the incorrect noise power gives inaccurate computation results in the update of the posterior probabilities. The smaller the $\sigma_{z}^{2}$ that the decision-maker employs, the quicker the growth in confidence level will be. As a consequence, when the channel is attacked, the decision-maker is misled by the unknown noise and it stops taking samples quickly and makes errors in process state detection. Nevertheless, if we compare the performances of SAC- and AC-based policies, we observe that performance degradation in the SAC policies is slightly smaller than that in the AC policies. This observation shows that the SAC algorithms have higher robustness in a noisy environment, and can be more effective against increased interference levels and jamming attacks.

We also test the algorithms with varying channel noise powers but under the assumption that this information is known to the decision-makers. We observe that if the noise variances become higher but are known to the decision-maker (as indicated by curves labeled without “+Jammer”), the accuracy levels are maintained by taking more observations from the sensors, which in turn leads to growth in both stopping time and total sensing cost.

#### 5.2.3. Cost Control

**Figure 8:**
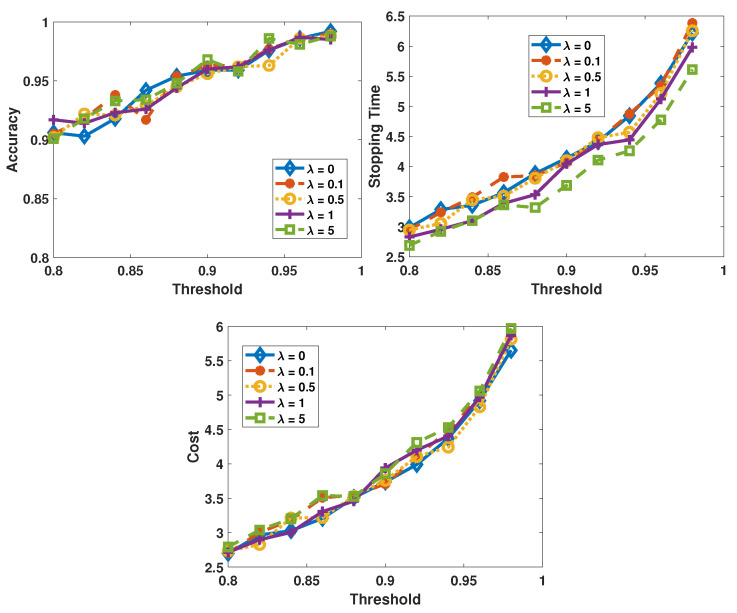
Performance of the SAC algorithm when the λ varies from 0 to 5, $\pi_{\mathrm{upper}}$ is varied from 0.8 to 0.98, and $\sigma^{2}=0.05$.

**Figure 9:**
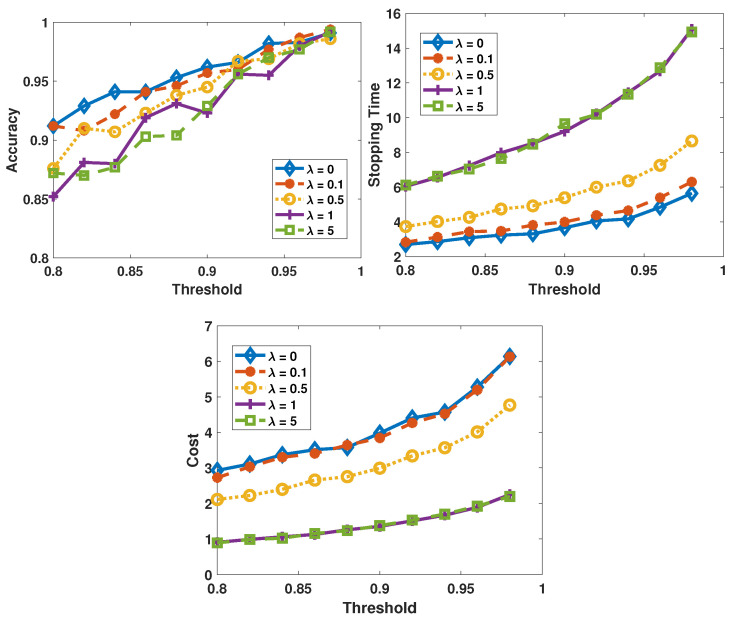
Performance of the AC algorithm when λ varies from 0 to 5, $\pi_{\mathrm{upper}}$ is varied from 0.8 to 0.98, and $\sigma^{2}=0.05$.

We first consider the cost function without a predefined average cost. It should be noted that, in the experiments, sensor 1 is the least costly but also the least reliable sensor, while sensor 3 is the most reliable sensor with the highest probing cost. As shown in the previous experiments, there is no obvious difference in the performance between the LLR reward-based policies and the entropy reward-based policies. So, we only demonstrate the performance of the LLR reward-based policies in experiments in this subsection. The performance metrics are studied as functions of $\pi_{\mathrm{upper}}$. Furthermore, the performances achieved by the same algorithm but with different values of λ are grouped. In Figure 8 and Figure 9, the performances of the SAC algorithm and the AC algorithm are plotted, respectively.

**Figure 10:**
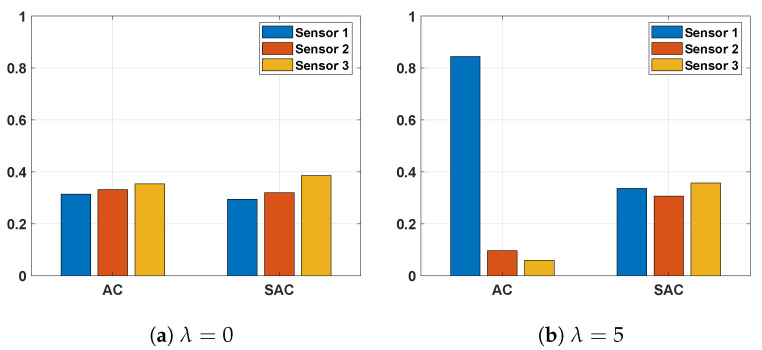
Distribution of sensor selection; $\pi_{\mathrm{upper}}=0.98,\sigma_{z}^{2}=0.6.$.

Obviously, the AC-based algorithm is more sensitive to the change in λ: when the value of λ increases, the average total cost decreases. The intuitive reasoning for this observation is that when the influence of cost is more pronounced in the reward due to higher weight λ, the AC-based algorithm tends to select the sensors with lower cost more frequently. Consequently, the more reliable sensors are selected less frequently, and therefore the decision accuracy decreases and the stopping time increases. As to the SAC-based decision-makers, the performance varies slightly as λ changes because the SAC-based algorithm prefers the most reliable sensor more frequently. To verify this, in Figure 10 we plot the fractions of samples coming from each sensor. Comparing the two figures, we find that when there is no cost (λ=0, in Figure 10a), the two algorithms’ policies are similar and the probabilities of selecting each sensor are close to each other. However, when there is a large λ (in Figure 10b), the AC algorithm selects sensor 1 with a probability as high as 0.84, whereas the SAC algorithm still selects sensor 3 with the highest probability even though the probability of selecting sensor 1 increases.

The two algorithms have different reactions to the change in λ because of the different strategies that are employed by them. The SAC algorithm is an off-policy maximum entropy DRL algorithm. To better explore the environment, the SAC algorithm favors a stochastic policy and aims at obtaining a more spread-out distribution in action probabilities. As to the AC algorithm, a more deterministic policy is pursued, which means that in a specific state, the AC algorithm only considers one action as the optimal solution. The above-mentioned characteristic of the SAC algorithm makes it more stable and robust in diverse settings and have less risk in overfitting to any local optimums. In this test case, the AC algorithm changes its policy dramatically as the λ changes. Compared to the AC algorithm, the SAC algorithm is more robust, maintaining strong performance over a wider range of λ values.

**Figure 11:**
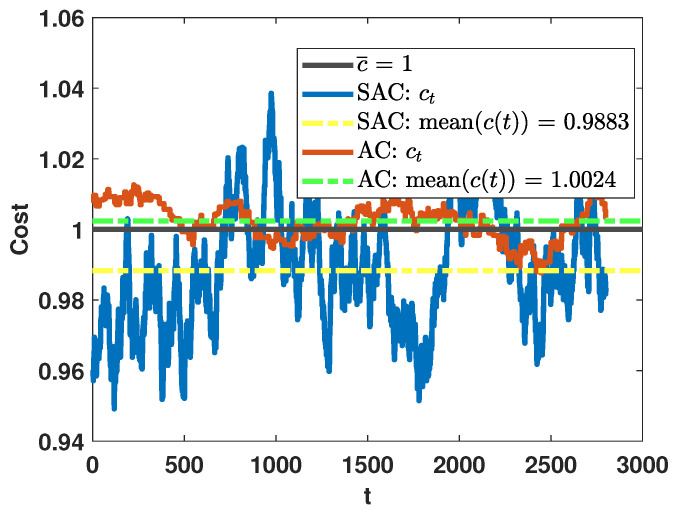
Performance in terms of cost, when the designed average cost is set as $\bar{c}=1$.

To take advantage of the more stochastic policy, we set a designed average cost $\bar{c}=1$ and feed back the corresponding cost to the decision-maker. In Figure 11, for the two DRL-based algorithms, we plot the moving average of cost $c_{t}=\frac{1}{W}\sum_{\tau =t-W+1}^{t}c\left(\tau \right)$, where the window size *W* is set to be 100. We observe that the moving average costs of both the proposed SAC algorithm and AC algorithm vary in a small range around the designed average cost. The difference is that the moving average cost of the SAC algorithm fluctuates in a wider range but the overall average cost is below the designed average cost, while the moving average cost achieved by the AC algorithm fluctuates in a narrower band but the overall average cost exceeds the designed average cost. In Table 4, we provide the corresponding detection accuracy and stopping time achieved by the two algorithms. In terms of both performance metrics, the SAC algorithm shows advantages over the AC algorithm. This is because the SAC algorithm is more flexible in the selection of actions, so it can take better advantage of selecting the most reliable sensor intermittently to ensure accuracy and reduce the detection delay at the same time.

#### 5.2.4. Byzantine

In this subsection, we consider the scenario in which the decision-maker operates in the presence of Byzantine sensors. It is assumed that at the beginning of each episode, there is an attacker randomly deciding whether to attack an honest sensor to make it a Byzantine or not. It is equally likely for the attacker to select any one from the three sensors or decide not to attack. Once the decision is made, the state of the sensors (honest or Byzantine) will remain fixed during the entire episode. Furthermore, as noted before, we assume there is an anchor node which is always honest and the anchor node obtains a record of the features from the training process of the GAN detector.

**Figure 12:**
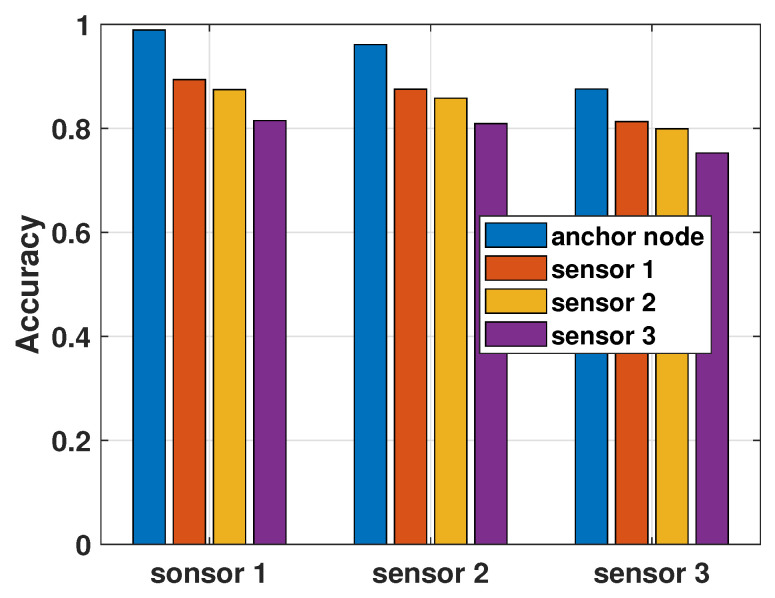
Accuracy of the GAN-based detector, when $\sigma_{z}^{2}=0.1$.

In Figure 12, we show the performance of the proposed GAN-based Byzantine detector in terms of detection accuracy. Since each sensor has its own noise power, the accuracy of different <suspected sensor, reference sensor> pairs will be different. Here, we consider all possible combinations of <suspected sensor, reference sensor> pairs. It should be noted that we also provide the accuracy achieved by the <sensor *i*, sensor *i*> pairs for i=1,2,3. In our aforementioned assumptions, the sensors cannot be the reference sensors for themselves. Therefore, in such sensor pairs, we assume that the suspected sensor and the corresponding reference sensor are identical but the states of the two sensors are independent. We only consider this situation in the test of the GAN detector’s accuracy, and in the detection of the process state, the suspected sensor and the reference sensor must be two different sensors. We notice in the figure that employing the anchor node as the reference sensor always achieves the highest accuracy. Furthermore, it can also be observed that noise power at the suspected sensor and the reference sensor is inversely proportional to the accuracy.

**Figure 13:**
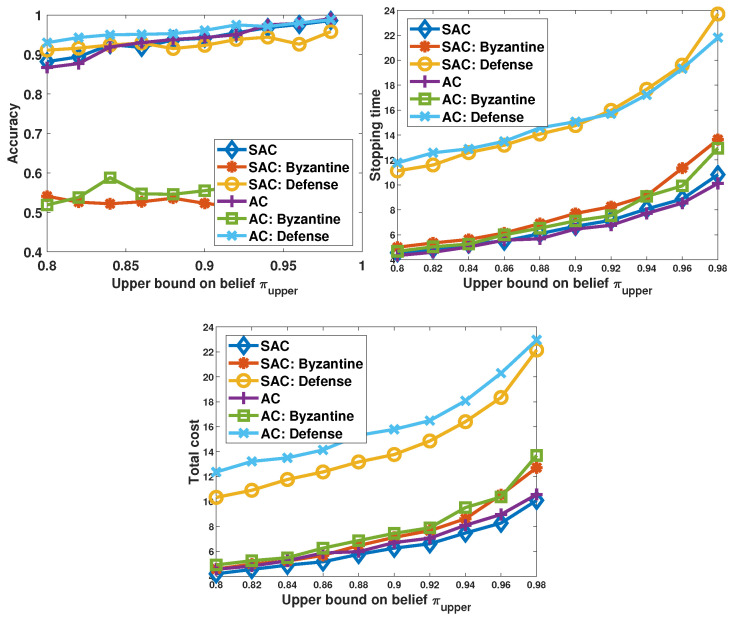
Performance of the decision-makers working in the absence of Byzantine attacks, with the presence of Byzantine attacks, and with the presence of both Byzantine attack and a GAN-based Byzantine detector when the λ=0, and $\sigma_{z}^{2}=0.1$.

With the pre-trained GAN-based detector, we investigate the performance of the defense strategy. When the sensors are employed as the reference sensors, the detection accuracy actually refers to that achieved by the trigger. Since the reference sensors are also noisy sensors, there is a probability that the samples from the reference sensors are distorted. To improve the accuracy of the trigger in the testing phase, instead of comparing the suspected sensor to only one reference sensor, we take all available reference sensors into consideration. Specifically, the sample from the current suspected sensor is compared with samples from all available reference sensors, and each comparison result is a decision on whether the suspected sensor is a Byzantine. Then, the majority decision is the final decision on the identity of the suspected sensor. With this “trigger–anchor” two-level detection, the Byzantines are identified and the corresponding samples are eliminated. In Figure 13, we plot the performance achieved in three cases. Specifically, “SAC/AC” refers to the situation in which the corresponding decision-maker works in the absence of Byzantine attacks, “SAC/AC:Byzantine” stands for the case in which the decision-maker operates in the presence of Byzantine attacks but there is no defense strategy, and “SAC/AC:Defense” denotes the scenario in which the decision-maker works in the presence of Byzantine attacks but employs the attack detection and defense strategy.

Considering the accuracy of detecting the process state, we observe that the proposed defense strategy can successfully recover the performance to the level achieved when no Byzantine attacks are executed. According to the decisions made by the GAN-based detector, the samples from the sensors which are labeled as Byzantine are removed. Therefore, to obtain sufficient information to reach the stopping criteria, more probing steps are taken. Consequently, there are obvious increases in both the stopping time and total cost. It can also be observed that with the GAN-based detector, the accuracy achieved by the “AC:Defense” scheme is higher than the “AC” scheme. This is because in the AC scheme, if sensor observations experience large noise levels that lead to quantization into incorrect states, these misleading quantized states are still transmitted to the decision-making agent and are utilized in the state detection. However, GAN-based Byzantine detectors identify and eliminate any misleading observations (due to Byzantines or high noise levels) in “AC:Defense”, resulting in improved accuracy levels.

## 6. Conclusions

In this work, we have proposed a soft actor–critic (SAC)-based reinforcement learning framework to address the reliable and fast state detection problem with a sensing cost control. First, we have modeled the sensor probing mechanism in the presence of two-level noise (i.e., noise in sensing and noise in the communication link between the sensor and the decision-making agent) and formulated the problem. We have developed the SAC-based algorithm with two types of rewards and two types of cost functions to reflect the objective and costs in the considered setting. Subsequently, we have considered the random Byzantine attacks on the sensors and designed a GAN-based agent to identify the Byzantine sensors. To evaluate the performance, we have considered three metrics: accuracy, stopping time, and total cost. In the experiments, we have compared the proposed SAC framework with the conventional actor–critic (AC) algorithm as well as fixed sensor-scheduling schemes.Via simulation results, we have demonstrated that the proposed SAC agent can be more robust in different test cases and the proposed GAN detector is able to identify the Byzantines with high accuracy and help to recover the detection performance achieved in the absence of Byzantine attacks.

## Figures and Tables

**Table 1:** Configuration of soft actor–critic algorithm.

	Policy Network	Q Network ^1^	Value Network
Input layer	200 neurons + ReLU	(200 + 200) neurons + ReLU	200 neurons + ReLU
Hidden layer	200 neurons + ReLU 200 neurons + ReLU 200 neurons + ReLU	200 neurons + ReLU 64 neurons + ReLU	200 neurons + ReLU 100 neurons + ReLU
Output layer	($2^{N}-1$) neurons + softmax	1 neuron	1 neuron
Learning rate	$e^{-5}$	$e^{-4}$	$e^{-4}$

^1^ Since both the observation and the action are taken as inputs of the Q network, the two components are loaded to the neural network through separate entries. Then, the extracted features of the observation and action will be merged in the second layer, and an estimated soft Q value will be given at the output layer.

**Table 2:** Configuration of conventional actor–critic algorithm.

	Actor	Critic
Input layer	200 neurons + ReLU	200 neurons + ReLU
Hidden layer	100 neurons + ReLU	100 neurons + ReLU
Output layer	($2^{N}-1$) neurons + Softmax	1 neuron
Learning rate	$5e^{-5}$	$1e^{-4}$

**Table 3:** Configuration of GAN.

	Generator	Discriminator
Input layer	128 neurons + ReLU	128 neurons + tanh
Hidden layer	128 neurons + ReLU 256 neurons + ReLU 256 neurons + ReLU	128 neurons + tanh 64 neurons + tanh
Output layer	(feature size) neurons	1 neuron + sigmoid
Learning rate	$e^{-5}$	$5e^{-3}$

**Table 4:** Performance of learning-based decision-makers with the designed average cost set to be 1.

	Accuracy	Stopping Time
SAC	0.9760	4.6020
AC	0.9630	5.5370

## Data Availability

The data supporting the conclusions of this article will be made available by the authors on request.
